# Increased SERPINA3 Level Is Associated with Ulcerative Colitis

**DOI:** 10.3390/diagnostics11122371

**Published:** 2021-12-16

**Authors:** Jingwei Zhang, Wei Wang, Shenglong Zhu, Yongquan Chen

**Affiliations:** 1 Wuxi School of Medicine, Jiangnan University, Wuxi 214122, China; 7160112076@vip.jiangnan.edu.cn (J.Z.); 7202808005@stu.jiangnan.edu.cn (W.W.); 8201910195@jiangnan.edu.cn (Y.C.); 2 School of Food Science and Technology, Jiangnan University, Wuxi 214122, China; 3 Wuxi Translational Medicine Research Center and Institute of Translational Medicine, Jiangnan University, Wuxi 214122, China

**Keywords:** ulcerative colitis, *SERPINA3*, biomarker

## Abstract

Ulcerative colitis (UC) is a recurrent, chronic intestinal disease that is currently incurable. Its pathogenesis remains to be further understood. Therefore, seeking new biomarkers and potential drug targets is urgent for the effective treatment of UC. In this study, the gene expression profile GSE38713 was obtained from the GEO (Gene Expression Omnibus) database. Data normalisation and screening of the differentially expressed genes (DEGs) were conducted using R software, and gene ontology (GO) enrichment was performed using Metascape online tools. The PubMed database was used to screen new genes that have not been reported, and *SERPINA3* was selected. The correlation between *SERPINA3* and other inflammatory factors was analysed by Spearman correlation analysis. Finally, colitis model mice and an in-vitro model were established to validate the function of the *SERPINA3* gene. *SERPINA3* gene expression was markedly increased in UC patient samples, colitis models and in-vitro models and showed an association with other inflammatory factors. ROC analysis indicated that *SERPINA3* could represent a potential biomarker of active UC. Additionally, silencing *SERPINA3* in an in-vitro intestinal epithelial inflammatory model significantly decreased the mRNA level of inflammatory factors. This study provides supportive evidence that SERPINA3 may act as a key biomarker and potential drug target in UC treatment.

## 1. Introduction

Ulcerative colitis (UC) is a chronic inflammatory gastrointestinal disorder [1] characterised by manifestations such as rectal bleeding, diarrhoea, abdominal pain, anaemia, and loss of body weight, which seriously attenuates the quality of life of patients [2,3]. In addition, long-term or even indefinite drug maintenance therapy imposes a substantial economic burden on patients [4].

Currently, the treatment of UC remains a challenge to clinicians [5]. The major pharmacological therapies for UC include corticosteroids, anti-inflammatory agents, and biologics [6]. Anti-inflammatory agents, such as 5-aminosalycerates (5-ASAs), have been the mainstay for the treatment of mild-to-moderate UC [7]. Although 5-ASAs are safe, their prolonged administration causes many side effects, such as headache, diarrhoea, nausea, interstitial nephritis, and hepatitis [8,9]. Corticosteroids are the mainstay of treatment for moderate to severe forms of UC [10]. However, corticosteroid use also creates side effects, including osteopenia, avascular necrosis, and mood changes [11,12]. Biologics alleviate UC by suppressing the inflammatory response [13]. However, a proportion of patients do not respond to biologics therapy or become intolerant or lose benefits [14,15,16]. Additionally, biologics are expensive, causing a tremendous economic burden for patients and medical care systems [17]. Therefore, identification of the key upstream regulatory gene is warranted for UC treatment.

Gene microarray technology has been used to analyse the molecular basis of many diseases [18]. Comprehensive and systemic analysis through gene microarray provides significant support to develop effective diagnosis and treatment strategies [19]. Currently, microarray technology has been widely used to elucidate disease progression and determine disease prognosis [20]. Presently, there exist studies mining UC-associated genes by public gene set databases. Previous studies have focused mainly on genes that have a close association with UC. In addition, the lack of experimental validation is another deficiency in these studies [21,22,23,24].

In this study, we performed multiple bioinformatics approaches to analyse UC gene microarray chip data and further analysed the clustered differentially expressed genes based on the Metascape online database. Next, we used the PubMed online database and selected *SERPINA3*, which has never been reported in UC. SERPINA3, also known as alpha-1 antichymotrypsin, acts as an inhibitor of several serine proteases. Insufficient serpin regulation causes excessive or prolonged cathepsin G activity, ultimately leading to tissue damage [25]. Previous studies have shown that SERPINA3 may act as a potential biomarker in several inflammatory-related diseases such as neurodegenerative diseases, cardiovascular diseases, and renal inflammatory diseases [26,27,28]. Accordingly, we further investigated the potential prognostic value of SERPINA3 and report on its association with UC, and ROC (receiver operator characteristic curve) analysis showed that SERPINA3 is a potential biomarker of active UC. The *SERPINA3* mRNA level was markedly increased in the mouse colitis model and human intestinal epithelial cell inflammatory model. Silencing *SERPINA3* in intestinal epithelial cells significantly attenuated the mRNA level of inflammatory factors. This study indicates that SERPINA3 is a new potential biomarker and therapeutic target of UC.

## 2. Materials and Methods

### 2.1. Microarray Data

The microarray data were obtained from the GEO database (https://www.ncbi.nlm.nih.gov/geo/) (accessed on 1 October 2021). The gene expression profile of GSE38713 was performed to select new potential genes, and the dataset from GSE36807 was selected for validation of gene expression of new potential genes. The microarray data of GSE38713 were obtained from GPL570 platforms, which contain a total of 43 intestinal mucosa samples: 13 healthy controls, 8 inactive UC, and 22 active UC samples. The microarray data of GSE36807 were from GPL570 platforms, which contain a total of 35 intestinal mucosa samples: 7 healthy controls, 15 UC, and 13 CD samples.

### 2.2. Identification of Differentially Expressed Genes (DEGs) in UC

The raw data of GSE38713 were downloaded from GEO as MINiML files. The extracted data were normalised and processed by log2 transformation. Probes were converted to gene symbols according to the GLP570 platform annotation information of the normalised data. An empirical Bayes method was used to select significant DEGs between UC samples and normal samples based on the “limma” package of Bioconductor (R software, version: 3.4). The Benjamin and Hochberg false discovery rate (FDR) method was used to correct the adjusted *p* value and correct the occurrence of false-positive results. FDR <0.05 and log (fold change) >1 or <−1 were defined as the thresholds of DEGs. The box plot and PCA graphs were drawn by the R software package “ggplot2”. The heatmap is displayed by the R software package “pheatmap”.

### 2.3. Gene Enrichment Analysis

Metascape online databases (https://metascape.org/) (accessed on 1 October 2021) support statistical analysis and visualisation of functional profiles for genes and gene clusters and were used to conduct DEG gene ontology analyses. DEGs were divided into two groups: the up DEGs and the down DEGs, and then they were analysed throughout Metascape.

### 2.4. Candidate Gene Validation

The candidate gene expression was validated by GSE36807. Gene correlation analysis was performed between candidate genes and inflammatory genes. ROC analysis was applied to evaluate the predictive power of the candidate gene. Both gene correlation analysis and ROC analysis curves were drawn by GraphPad Prism 8.0 (GraphPad Software, Inc., San Diego, CA, USA).

### 2.5. Establishment of the Mouse Model of Colitis

Thirteen 8-week-old male C57BL/6J mice were purchased from Shanghai SLAC Laboratory Animal Co., Ltd. (Shanghai, China), and were housed in a specific pathogen-free (SPF) environment. Mice were subjected to light for a 12 h/darkness cycle, a normal diet, and water with an ambient temperature of 24–26 °C and humidity of 50% to 60%. After one week of adaptive feeding, the mice were modelled from the second week. The colitis model was induced by using dextran sodium sulfate (DSS, colitis grade, 0216011080, MP Biomedicals, Santa Ana, CA, USA). Mice were divided into a control group (n = 5) and a DSS group (n = 8). Colitis was induced by adding 2.5% DSS to the drinking water of the animals for 8 days. During the experiment, body weight was recorded every day.

### 2.6. Colitis Assessment

Eight days after modelling, the colon was removed from sacrificed mice. A 1-cm portion of the distal colon was harvested, and one 0.5-cm colon was used for paraffin sections. In brief, colon tissues were fixed with 4% neutral formaldehyde solution for 48 h, and then paraffin sections (5 μm) were prepared by dehydration, transparency, wax dipping, and embedding. Finally, the sections were stained with haematoxylin-eosin (HE) reagents. The histopathological alterations were scored according to the method of Dieleman et al. [29]: 0, no inflammation and mucosal damage; 1, inflammatory cell infiltration into the mucosal layer and loss of basal 1/3 of crypts; 2, inflammatory cell infiltration into the submucosa and loss of basal 2/3 of crypts; 3, inflammatory cell infiltration into the muscularis mucosae and entire crypt loss; and 4, entire epithelial and crypt damage. Another 0.5-cm colon was used for quantitative real-time polymerase chain reaction (qRT-PCR) to test the transcript levels of *SERPINA3* and other inflammatory factors.

### 2.7. Cell Culture

HT29 human intestinal epithelial cells were purchased from the Cell Bank of Type Culture Collection of the Chinese Academy of Sciences (Shanghai, China). Cells were cultured in DMEM (Gibco) with 10% foetal bovine serum (04-001-1ACS, Biological Industries, Kibbutz Beit-Haemek, Israel) at 37 °C in the presence of 5% CO_2_. TNFα (10 ng/mL, Z01001, GenScript Biotech, Nanjing, China) was used in cell culture with HT29 cells for 12 h to mimic an inflammatory background [30].

### 2.8. SERPINA3 Gene Silencing

Before small interfering RNA (siRNA) transfection, the cells were plated to obtain a next-day confluency of 50%. On the day of transfection, cells were transfected with 50 nM siRNA using JetPrime (101000046, Polyplus, Shanghai, China) transfection reagent. Sequences of SERPINA3 siRNA are: UGGAAUGCAAGCUGGAUGCCUTT. Universal negative control siRNA (A06001, GenePharma, Shanghai, China) was used as a control.

### 2.9. RNA Isolation and qRT-PCR

Colon or cell total RNA was isolated with the MolPure^®^ TRIeasy™ Plus Total RNA Kit (19211ES60, Yeasen Biotech, Shanghai, China) according to the manual instructions. cDNA was synthesised with a HiScript III 1st Strand cDNA Synthesis Kit (R312, Vazyme Biotech, Nanjing, China) according to kit instructions. The human primer sequences used for qPCR were as follows: *SERPINA3*: CCTGAAGGCCCCTGATAAGAA (forward, 5′–3′), GCTGGACTGATTGAGGGTGC (reverse, 5′–3′); *CXCL8*: ACTGAGAGTGATTGAGAGTGGAC (forward, 5′–3′), AACCCTCTGCACCCAGTTTTC (reverse, 5′–3′); *CCL2*: CAGCCAGATGCAATCAATGCC (forward, 5′–3′), TGGAATCCTGAACCCACTTCT (reverse, 5′–3′); *β-ACTIN*: CATGTACGTTGCTATCCAGGC (forward, 5′–3′), CTCCTTAATGTCACGCACGAT (reverse, 5′–3′). The mouse primer sequences used for qPCR were as follows: *Serpina3*: ATTTGTCCCAATGTCTGCGAA (forward, 5′–3′), TGGCTATCTTGGCTATAAAGGGG (reverse, 5′–3′); *IL1b*: CTGAACTCAACTGTGAAATGC (forward, 5′–3′), TGATGTGCTGCTGCGAGA (reverse, 5′–3′); *IL6*: CTCTGCAAGAGACTTCCATCCAGT (forward, 5′–3′), GAAGTAGGGAAGGCCGTGG (reverse, 5′–3′); *Tnf*: AGGGTCTGGGCCATAGAACT (forward, 5′–3′), CCACCACGCTCTTCTGTCTAC (reverse, 5′–3′); *β-actin*: TGTTACCAACTGGGACGACA (forward, 5′–3′), CTGGGTCATCTTTTCACGGT (reverse, 5′–3′). A total volume of 20 µL was used for the qRT-PCR containing 50 ng template cDNA, 8 pmol forward primer and reverse primer, and 10 µL Hieff^®^ qRT-PCR SYBR Green Master Mix (11201ES03, Yeasen Biotech, Shanghai, China). The amplification program was as follows: (1) 95 °C for 5 min, (2) 95 °C for 10 s, 60 °C for 30 s followed by 40 cycles.

### 2.10. Western Blotting

Western blotting was performed as previously reported [31]. Briefly, tissues or cells were collected and lysed using RIPA buffer (P0013B, Beyotime, Biotechnology, Shanghai, China) with protease inhibitor cocktail (HY-K0010, MCE, Shanghai, China). Afterwards, samples were exposed to 5 cycles of 5 s ultrasound treatments and centrifuged at 12,000× *g* in a refrigerated centrifuge for 10 min. Samples were equalised according to the protein concentration and separated with SDS-PAGE. The proteins were electrically transferred to PVDF membranes (ISEQ00010, Millipore, Shanghai, China) followed by blocking the PVDF membranes for 1 h at room temperature with 5% skim (232100, BD, Sparks, MD, USA) in TBST. The membranes were incubated overnight at 4 °C with primary antibodies (A1021, anti-SERPINA3, ABclonal, Wuhan China; AC026, anti-β-ACTIN, ABclonal, Wuhan, China). The following day, membranes were washed 3 times in TBST, and incubated with HRP-conjugated secondary IgG antibody (AS014, ABclonal, Wuhan, China) for 1 h at room temperature. Before imaging, the membranes were washed with TBST 3 times and ECL reagent kit (WBKLS0500, Millipore, Shanghai, China) was used for detection of expressed proteins.

### 2.11. Statistical Analysis

The data are presented as the mean ± standard deviation (SD). Student’s *t*-test was used to analyse the significant differences among different groups. Statistical significance was considered when the *p*-value <0.05.

## 3. Results

### 3.1. DEGs between UC and Health Control

**Figure 1:**
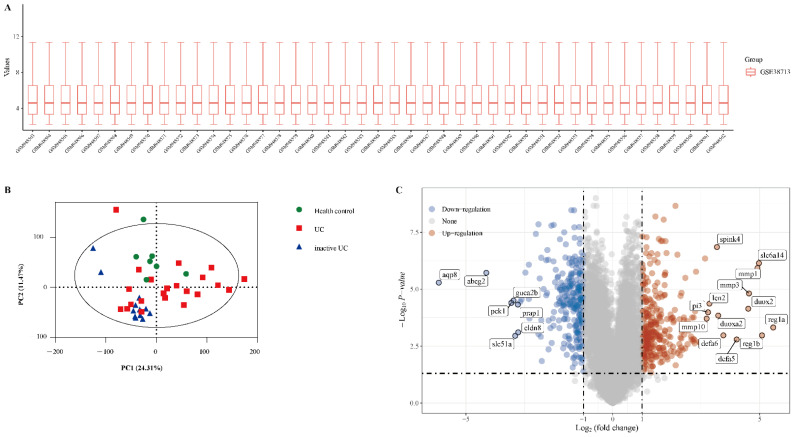
Identification of differentially expressed genes (DEGs) in the dataset GSE38713. (**A**) The extracted data in GSE38713 were normalised and processed by log2 transformation. (**B**) Principal component analysis (PCA) of dataset GSE38713. Three groups of samples (healthy control, green; UC, red; inactive UC, blue) are shown in the PCA plot. (**C**) The volcano plot of the GSE38713. The red data points represent the upregulated genes (log2FC >1, FDR <0.05) and the blue data points represent the downregulated genes (log2FC <−1, FDR <0.05).

The gene expression dataset GSE38713 includes 13 healthy control samples and 30 UC samples (8 inactive UC and 22 active UC). As shown in Figure 1A, the data distributions were neat after background adjustment and normalisation. Next, all data were analysed using principal component analysis (PCA). PCA revealed that the three groups were relatively well separated (Figure 1B). We used the “limma” package to identify the DEGs in GSE38713 with FDR <0.05 and log (fold change) >1 or <−1. There were 560 DEGs in the GSE38713 dataset, including 309 upregulated genes and 251 downregulated genes. DEGs were visualised using a volcano plot (Figure 1C), and the DEGs with significant fold changes were labelled in the plot.

### 3.2. SERPINA3 Is Significantly Increased in UC Patients

**Figure 2:**
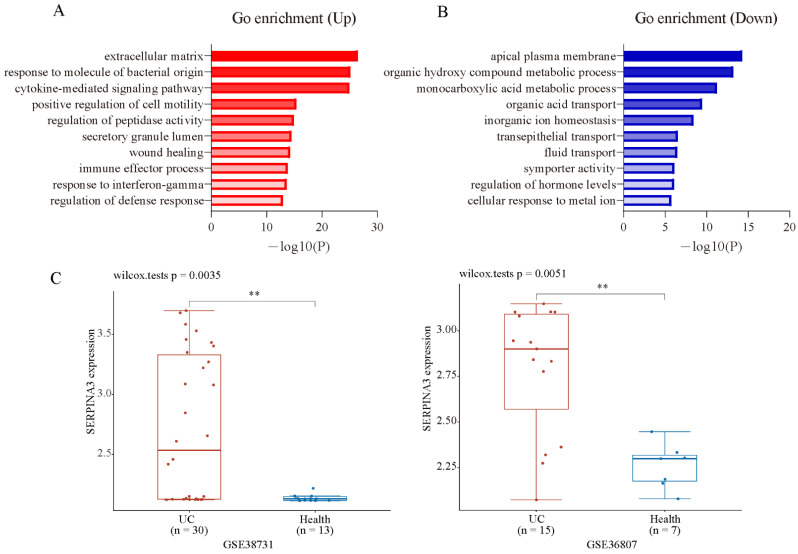
GO enrichment analysis and candidate gene validation. Gene ontology analysis of the up- (**A**) and downregulated (**B**) DEGs was conducted using Metasacape online tools (*p* < 0.01). (**C**) Validation of the candidate gene *SERPINA3* in GSE38713 and GSE36807. ** *p* < 0.01.

To analyse the biological classification of DEGs, the Metascape online database was used for gene enrichment analysis. The upregulated and downregulated DEGs were uploaded to Metascape. The upregulated DEGs were enriched mainly in inflammation and extracellular matrix-related terms (Figure 2A). The downregulated DEGs were enriched mainly in the transportation and metabolism of organic and inorganic small molecules (Figure 2B). The top three enriched GO terms, depending on the −log10 *p* value, are presented in Table 1 (*p* < 0.01). To screen the candidate genes that may be key biomarkers and potential drug targets in UC treatment, we focused on the GO term “extracellular matrix”, which has the largest −log10 *p*-value in the enrichment of up DEGs. There were 47 genes significantly enriched in this GO term, which contained some family genes, such as matrix metalloproteinase (MMP) family genes (*MMP1*, *MMP3*, *MMP7*, *MMP9*, *MMP10*, *MMP12*), collagen (COL) family genes (*COL1A1*, *COL1A2*, *COL3A1*, *COL4A1*, *COL5A2*, *COL6A3*, *COL15A1*), serine proteinase inhibitor (SERPIN) family genes (*SERPINA3*, *SERPING1*, *SERPINB5*), and claudin (CLDN) family genes (*CLND1*, *CLDN2*). Next, PubMed (up to October 2021) was used to screen new candidate genes, and the following terms were used to screen: ** AND (colitis OR UC OR IBD OR ulcerative colitis OR inflammatory bowel disease), and ** indicates one gene that is included in the GO term “extracellular matrix”. After a literature search and gene screening, a total of 18 genes were selected. The associations of the selected genes with UC that were not previously reported were ranked according their log2FC (Table 2). As the fold change of *SERPINA3* was the largest, *SERPINA3* was selected for further study. Next, we validated the expression of *SERPINA3* in GSE38713 and GSE36807. The difference in *SERPINA3* expression between UC samples and healthy control samples was determined by the Wilcoxon rank-sum test. As shown in Figure 2C, *SERPINA3* gene expression was significantly upregulated in the intestinal mucosa of UC patients in both GSE38713 and GSE36807.

### 3.3. SERPINA3 Is a Potential Biomarker for the Active UC

**Figure 3:**
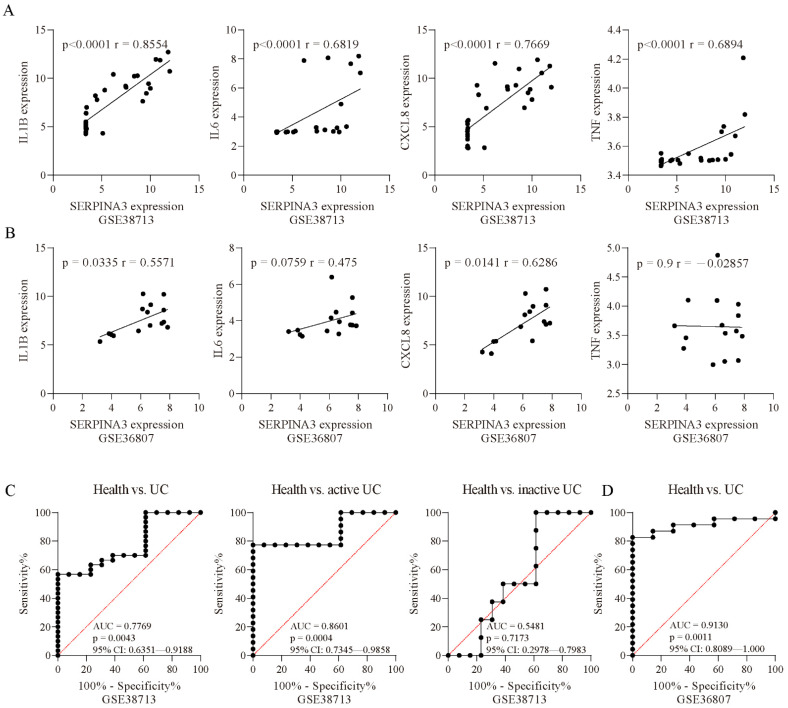
Correlation analysis. Correlation analysis of *SERPINA3* and other inflammatory factors (*IL1B*, *IL6*, *CXCL8*, and *TNF*) in the datasets (**A**) GSE38713 and (**B**) GSE36807. Spearman’s correlation coefficient r and the *p* value for each gene pair are indicated in each figure. ROC curve of *SERPINA3* gene expression for UC diagnosis. The ROC curve data are derived from the datasets (**C**) GSE38713 and (**D**) GSE36807. In GSE38713, the UC samples were subdivided into two groups: inactive UC and active UC. The area under the curve (AUC), *p* value, and 95% confidence interval (95% CI) are shown in each figure.

To further examine whether *SERPINA3* gene expression was associated with biomarkers of inflammation, we performed correlation analyses. In the UC samples of GSE38713, *SERPINA3* expression was positively correlated with *IL1B* (*p* < 0.0001, r = 0.8554), *IL6* (*p* < 0.0001, r = 0.6819), *CXCL8* (*p* < 0.0001, r = 0.7669), and *TNF* (*p* < 0.0001, r = 0.6894) (Figure 3A). In the UC samples of GSE36807, *SERPINA3* expression showed a positive correlation with *IL1B* (*p* = 0.0335, r = 0.5571) and *CXCL8* (*p* = 0.0141, r = 0.6286) and was not correlated with *IL6* (*p* = 0.0759, r = 0.475) or *TNF* (*p* = 0.9, r = −0.02857) (Figure 3B). These findings suggested that *SERPINA3* was correlated with inflammation in UC. Next, ROC curves were used to determine whether *SERPINA3* exhibited diagnostic significance for UC, and the area under the curve (AUC) value of the ROC curve reflects the quality of the ROC curve. In the GSE38713 dataset, the AUC for *SERPINA3* was 0.7669. Considering that the GSE38713 dataset contains active UC samples and inactive UC samples, these two types of samples were separated for further analysis. For the active UC samples, the AUC for *SERPINA3* was 0.8601, and for the inactive UC samples, the AUC for *SERPINA3* was 0.5481 (*p* = 0.7173) (Figure 3C). Additionally, the GSE36807 dataset was used for further validation, and the AUC for *SERPINA3* was 0.913 (Figure 3D). These findings suggest that *SERPINA3* is a potential biomarker for active UC but not inactive UC.

### 3.4. Verification of SERPINA3 Function in Mice Model

**Figure 4:**
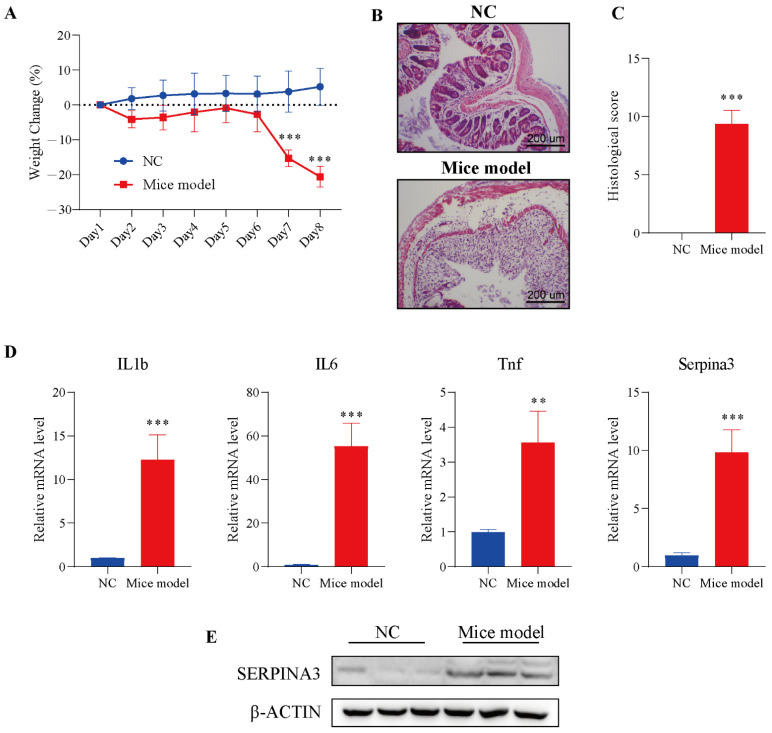
SERPINA3 was upregulated in the mouse colon after DSS treatment. Mice were divided into two groups: the control group (n = 5, NC) and the DSS treatment group (n = 8, mouse model). Mice received 2.5% DSS for 7 days. Throughout the DSS treatment period, daily body weight was recorded. At the end of the modelling day, the mice were sacrificed, and distal colon samples were used for paraffin sectioning and qRT-PCR detection. (**A**) Body weight change in mice during the experimental period. (**B**) HE staining of colon tissues from the control and DSS-induced colitis model mice. (**C**) Histological scores of colon tissues. (**D**) The mRNA levels of *IL1b*, *IL6*, *Tnf*, and *SERPINA3*. (**E**) SERPINA3 protein expression was detected by Western blot. ** *p* < 0.01, *** *p* < 0.001.

To verify the analysis results described above, we used a mouse colitis model to evaluate the expression of *SERPINA3* in colitis mice. Before verification, DSS was employed to establish the colitis model in mice. Mice were monitored for weight during the DSS treatment. On Day 7 of modelling, the DSS model group exhibited significant body weight loss, with the trend continuing through Day 8. In the control group, a slowly increasing trend for body weight emerged from Day 1 to Day 8 (Figure 4A). Next, intestinal sections of the control and DSS treatment groups were observed under microscopy. In the control group, HE staining results showed that the colonic mucosa was intact, and the intestinal epithelial cells and glands were arranged neatly, without significant pathological changes. 

Compared with the control group, the intestinal mucosa in the DSS treatment group showed severe morphological damage, including marked crypt destruction, heavy inflammatory cellular infiltrate, and extensive destruction of the mucosal layer (Figure 4B). Therefore, the histological score in the DSS treatment group was significantly higher than the histological score in the control group (Figure 4C). The results of the above experiments showed the successful establishment of the colitis model. Next, the levels of inflammatory factors (*IL1b*, *IL6*, *Tnf*) and *Serpina3* were analysed by qRT-PCR. The transcription levels of three inflammatory factors and *Serpina3* in the model group were significantly increased. In addition, the protein level of SERPINA3 was also markedly increased (Figure 4D,E). This result was consistent with the bioinformatics analysis above.

### 3.5. Silencing SERPINA3 Attenuated Inflammation Status in an In-Vitro Model

**Figure 5:**
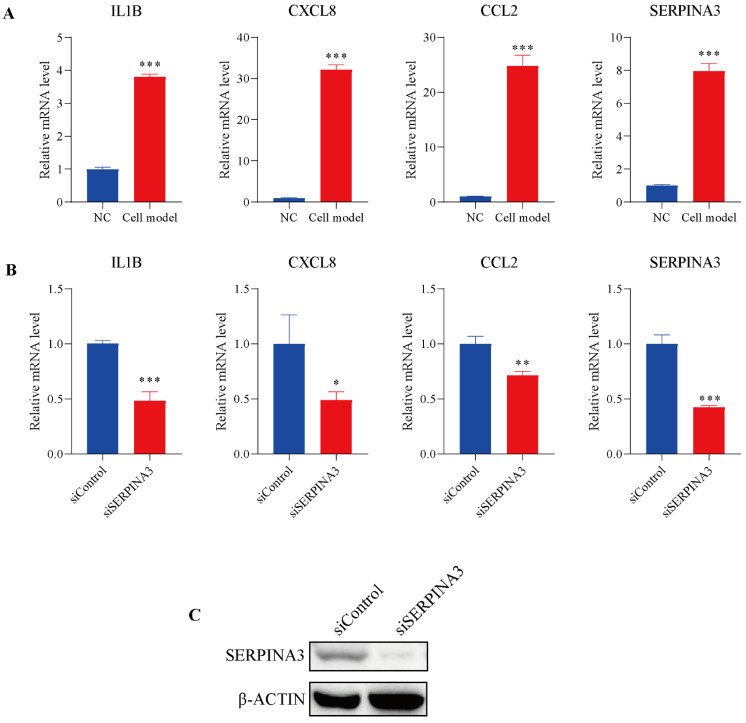
The potential role of SERPINA3 in an in-vitro UC model. HT29 intestinal epithelial cells were incubated with (cell model) or without (NC) TNFα (10 ng/mL) for 12 h to mimic inflammatory conditions in vitro. (**A**) After TNFα stimulation, cell RNA was isolated, and the transcription levels of *IL1B*, *CXCL8*, *CCL2*, and *SERPINA3* were detected by qRT-PCR. HT29 intestinal epithelial cells were transfected with control siRNA (siControl) or *SERPINA3* siRNA (siSERPINA3). Forty-eight hours post-transfection, all the groups were incubated with TNFα (10 ng/mL) for 12 h. After transfection and TNFα stimulation, cell RNA was isolated. (**B**) The transcription levels of *IL1B*, *CXCL8*, *CCL2* and *SERPINA3* were detected by RT-qPCR. (**C**) SERPINA3 protein expression was detected by Western blot. * *p* < 0.05, ** *p* < 0.01, *** *p* < 0.001.

To further study the potential role of *SERPINA3* in UC, HT29 intestinal epithelial cells were incubated with TNFα to mimic inflammatory conditions in vitro. As shown in Figure 5A, TNFα stimulated noticeable transcription levels of *IL1B*, *CXCL8*, and *CCL2*, and the mRNA levels of *SERPINA3* were also dramatically increased. Subsequently, *SERPINA3* was silenced for 48 h, and the cells were incubated with TNFα for 12 h. As shown in Figure 5B, silencing *SERPINA3* resulted in a marked decrease in the levels of *IL1B*, *CXCL8*, and *CCL2*. *SERPINA3* knock down efficiency was validated by Western blot (Figure 5C). These results showed that SERPINA3 plays a proinflammatory role in intestinal epithelial inflammation.

## 4. Discussion

The incidence of UC has been rising in recent years [32]. Currently, due to its complex aetiology, the major barriers to medicine in UC are the lack of biomarkers and therapeutic targets [33], and the molecular mechanisms driving the disease course and response to therapy in ulcerative colitis (UC) are not well understood. To improve our understanding of UC pathogenesis and provide new directions for clinical diagnosis, we applied multiple large bioinformatics approaches to analyse UC gene microarray chip data.

In this study, we analysed the DSE38713 dataset through bioinformatics analysis, and GO analysis showed that the extracellular matrix and the inflammatory process were significantly enriched. The top 1 GO term was “extracellular matrix”. Previous studies have shown that the degradation and formation of extracellular matrix are associated with intestinal damage during inflammation, indicating that extracellular matrix may play a critical role in the pathology of UC, which arouses our interest in exploring whether we will be able to find a new gene related to UC in this GO term.

To identify UC-associated genes that have never been reported before, the PubMed database was used to search and screen. A total of 18 genes were selected, and *SERPINA3* ranked first in terms of log2FC. SERPINA3 is a member of the serine protease inhibitor (SERPIN) superfamily. SERPINA3 has been reported to be upregulated in some inflammatory responses, such as cardiovascular inflammation and renal inflammation [34,35], but the role of SERPINA3 in UC has not been investigated. Several studies have shown that SERPINA3 can be found in plasma and urine, suggesting that SERPINA3 may be a useful non-invasive biomarker [36,37,38]. In this study, the correlation analysis showed that the expression of *SERPINA3* was associated with inflammatory factors, and ROC analysis suggested that *SERPINA3* was a potential biomarker of active UC. Additionally, the mRNA and protein levels of SERPINA3 were markedly increased in the inflamed colon. Previous in vivo studies found that SERPINA3 was markedly upregulated in the mice model of experimental autoimmune myocarditis and chronic pulmonary injuries [39,40]. These studies indicated that SERPINA3 could be a widely inflammatory biomarker. Given that SERPINA3 is a secretory protein, therefore, investigations on the correlation between UC and SERPINA3 protein levels in plasma or urine would be meaningful in the future.

The application of biologics has brought significant improvement in the management of UC [41,42]. Biologics, such as infliximab or adalimumab, have been used for the treatment of UC patents to alleviate symptoms [43]. Although biologic therapy exerts good therapeutic effects in a proportion of patients, approximately one-third of patients do not respond to them [42]. Additionally, patients who lose response to therapy may increase with longer periods of use [44]. A simple inflammatory target could be one reason for this phenomenon. The upstream targets of inflammatory signalling and multi-pathway inhibition are plausible solutions. Thus, uncovering the key upstream modulatory genes of inflammatory signals is warranted. In this study, we found that silencing *SERPINA3* in an in vitro model markedly decreased the mRNA level of inflammatory factors, indicating that *SERPINA3* may act as an upstream regulatory gene. With the development of monoclonal antibody therapy and gene therapy technology, targeting SERPINA3 may offer an innovative direction for UC therapeutic strategies.

## 5. Conclusions

In summary, we have uncovered a new function of *SERPINA3* and demonstrated the inhibitory effect of *SERPINA3* on the inflammation process in UC. Our study broadens the understanding of the pathogenesis of UC and provides potential therapeutic targets for the treatment of UC.

## Figures and Tables

**Table 1:** Top 3 GO terms and relative up and down genes.

**Up**				
**Go Term**	**Description**	**−Log10P**	**Count**	**Genes**
GO:0031012	extracellular matrix	26.52245	47	*SERPINA3*, *AEBP1*, *AGT*, *ANXA5*, *AZGP1*, *SERPING1*, *CHI3L1*, *COL1A1*, *COL1A2*, *COL3A1*, *COL4A1*, *COL5A2*, *COL6A3*, *COL15A1*, *CPA3*, *CTSH*, *DMBT1*, *ECM1*, *F3*, *LAMC1*, *LGALS1*, *MMP1*, *MMP3*, *MMP7*, *MMP9*, *MMP10*, *MMP12*, *PCOLCE*, *PF4*, *PI3*, *HTRA1*, *S100A8*, *S100A9*, *SLPI*, *SPARC*, *TGFBI*, *TIMP1*, *WNT5A*, *PXDN*, *TFPI2*, *SPARCL1*, *SPON2*, *SULF1*, *ANGPTL2*, *MXRA5*, *GREM1*, *CTHRC1*, *CAV1*, *CTSK*, *SERPINB5*, *PRDX4*, *SPINK5*, *CSRP2*, *SPAG4*, *CLDN2*, *CLDN1*, *OLFM4*
GO:0002237	response to molecule of bacterial origin	25.18266	38	*ASS1*, *C5AR1*, *CASP1*, *CSF2RB*, *CD55*, *DEFA5*, *DEFA6*, *DMBT1*, *FCGR2B*, *GJA1*, *CXCL1*, *CXCL3*, *HCK*, *IL1B*, *IL10RA*, *CXCL10*, *LCN2*, *LYN*, *CXCL9*, *NOS2*, *PF4*, *S100A8*, *S100A9*, *CXCL6*, *CXCL11*, *CXCL5*, *SELP*, *SLPI*, *SPARC*, *TLR2*, *WNT5A*, *CLDN1*, *LILRB2*, *SPON2*, *CXCL13*, *LY96*, *CD274*, *TNIP3*, *C3*, *C4BPA*, *C4BPB*, *CAV1*, *DEFB4A*, *IL7R*, *PI3*, *PLA2G2A*, *S100A12*, *SPINK5*, *REG4*, *IGLL5*, *COL1A1*, *SPP1*, *LRP8*, *RAMP3*, *AGT*, *CHI3L1*, *F3*, *MMP12*, *MNDA*, *POU2AF1*, *SLC7A5*, *SERPINB7*, *AIM2*, *SULF1*, *HEG1*, *IL33*, *BST2*, *CDH3*, *TRIB2*
GO:0019221	cytokine-mediated signalling pathway	24.97113	42	*TNFRSF17*, *CASP1*, *CAV1*, *CSF2RB*, *ECM1*, *F3*, *CXCL1*, *CXCL3*, *HCK*, *IL1B*, *IL1RN*, *IL7R*, *CXCR2*, *IL10RA*, *IL13RA2*, *CXCL10*, *CXCL9*, *MMP12*, *PF4*, *ROBO1*, *CCL11*, *CCL18*, *CCL24*, *CXCL6*, *CXCL11*, *CXCL5*, *WNT5A*, *LRP8*, *PXDN*, *IFITM1*, *OSMR*, *AIM2*, *LILRB2*, *IFITM3*, *CXCL13*, *IFITM2*, *TNFSF13B*, *DUOX2*, *ACKR4*, *PLVAP*, *CCDC3*, *IL33*, *SERPING1*, *C1R*, *C3*, *C4BPA*, *C4BPB*, *CD55*, *DEFA5*, *DEFA6*, *DEFB4A*, *DMBT1*, *FCGR2B*, *CFI*, *REG3A*, *PI3*, *POU2AF1*, *REG1A*, *REG1B*, *S100A9*, *S100A12*, *SLPI*, *SPON2*, *SPINK5*, *SPNS2*, *IGLL5*, *C5AR1*, *LYN*, *MMP9*, *PECAM1*, *S100A8*, *SELL*, *SELP*, *THY1*, *PLA2G7*, *MADCAM1*, *OLFM4*, *GREM1*, *AGT*, *FPR1*, *GNA15*, *PNOC*, *ADA2*, *PROK2*, *MRAP2*, *CTHRC1*, *CDH5*, *ITGA5*, *SPP1*, *TFF1*, *TIMP1*, *MANF*, *GMFG*, *CHN1*, *PLAU*
**Down**				
**Go Term**	**Description**	**−Log10P**	**Count**	**Genes**
GO:0016324	apical plasma membrane	14.3201	25	*CA4*, *CEACAM7*, *SLC26A2*, *P2RY1*, *ABCB1*, *PRKG2*, *SCNN1B*, *SLC1A1*, *SLC16A1*, *SLC22A4*, *SLC22A5*, *STX3*, *PLPP1*, *ABCB11*, *ABCG2*, *SLC23A1*, *NAALADL1*, *SLC17A4*, *CLCA4*, *CDHR5*, *RAB17*, *CYP4F12*, *PDZD3*, *TRPM6*, *SLC6A19*, *AQP8*, *LIMA1*, *CNGA1*, *GABRA2*, *PDE6A*, *PHLPP2*, *PSD3*, *CDHR1*
GO:1901615	organic hydroxy compound metabolic process	13.217	28	*ABAT*, *ADH1C*, *ADH6*, *CYP27A1*, *DDC*, *EPHX2*, *FMO5*, *HMGCS2*, *ITPKA*, *MAOA*, *P2RY1*, *PCK1*, *PRKG2*, *SCNN1B*, *SLC1A1*, *SULT1A2*, *VLDLR*, *PLPP1*, *ABCB11*, *OPN3*, *AMACR*, *NAAA*, *SULT1B1*, *LIMA1*, *RETSAT*, *CYP4F12*, *OSBPL1A*, *LDHD*, *CYP2B6*, *HSD17B2*, *DHRS11*
GO:0032787	monocarboxylic acid metabolic process	11.2724	27	*ABAT*, *ACADM*, *ACADS*, *ADH1C*, *ADH6*, *ENTPD5*, *CPT1A*, *CYP2B6*, *CYP27A1*, *EDN1*, *ETFDH*, *HPGD*, *PCK1*, *PDK2*, *PPARG*, *ABCB11*, *SLC4A4*, *LIAS*, *AMACR*, *NAAA*, *CYP2S1*, *UGT1A10*, *CYP4F12*, *UGT2A3*, *ACSF2*, *OSBPL1A*, *LDHD*, *SULT1A2*, *APOBEC3B*, *DDAH2*, *SULT1B1*, *APOBEC3A*, *PLCD1*, *PRDX6*, *PLA2G12B*, *FMO4*, *FMO5*, *AIFM3*, *EPHX2*, *PLPP1*, *B4GALNT2*, *RETSAT*

**Table 2:** Selected genes after literature search. Genes were ranked by log2FC.

Gene	log2FC	AveExpr	*p*-Value	adj. P Val.
*SERPINA3*	2.781464	5.339446	0.002387	0.014217
*CTHRC1*	2.752319	5.963112	0.000175	0.002244
*COL4A1*	2.062337	8.255427	0.00029	0.003163
*SPINK5*	1.807568	9.775712	0.002094	0.012913
*TGFBI*	1.751269	10.09177	1.87 × 10^−5^	0.000504
*SPAG4*	1.619358	5.398344	0.001235	0.008798
*SPON2*	1.586121	6.648104	0.001681	0.011077
*COL5A2*	1.578861	7.339977	0.001568	0.010548
*PXDN*	1.463779	6.636386	0.000362	0.003714
*AZGP1*	1.293119	5.853493	0.003716	0.019688
*PCOLCE*	1.284043	6.823738	0.006083	0.028299
*CTSH*	1.230996	9.696432	0.000707	0.005954
*LAMC1*	1.171772	7.556216	0.000159	0.002112
*ANGPTL2*	1.163704	5.229639	0.001209	0.008677
*MXRA5*	1.086506	8.460376	0.024482	0.080414
*AEBP1*	1.076301	7.03746	0.016754	0.0603
*CSRP2*	1.075397	7.034764	0.008392	0.036062
*CPA3*	1.063069	8.603432	0.031116	0.096113

## Data Availability

The data used to support the findings of this study are available from the corresponding author upon request.
